# New insights into leaf and fine‐root trait relationships: implications of resource acquisition among 23 xerophytic woody species

**DOI:** 10.1002/ece3.1794

**Published:** 2015-10-29

**Authors:** Fang Lan Li, Wei Kai Bao

**Affiliations:** ^1^ Key Laboratory of Mountain Ecological Restoration and Bioresource Utilization Chengdu Institute of Biology Chinese Academy of Sciences Chengdu China

**Keywords:** Belowground ecology, carbon balance, functional traits, interspecific correlation, nutrient economic, whole‐plant strategy

## Abstract

Functional traits of leaves and fine root vary broadly among different species, but little is known about how these interspecific variations are coordinated between the two organs. This study aims to determine the interspecific relationships between corresponding leaf and fine‐root traits to better understand plant strategies of resource acquisition. SLA (Specific leaf area), SRL (specific root length), mass‐based N (nitrogen) and P (phosphorus) concentrations of leaves and fine roots, root system, and plant sizes were measured in 23 woody species grown together in a common garden setting. SLA and SRL exhibited a strong negative relationship. There were no significant relationships between corresponding leaf and fine‐root nutrient concentrations. The interspecific variations in plant height and biomass were tightly correlated with root system size characteristics, including root depth and total root length. These results demonstrate a coordinated plant size‐dependent variation between shoots and roots, but for efficiency, plant resource acquisition appears to be uncoupled between the leaves and fine roots. The different patterns of leaf and fine‐root traits suggest different strategies for resource acquisition between the two organs. This provides insights into the linkage between above‐ and belowground subsystems in carbon and nutrient economy.

## Introduction

Leaves and fine roots are active organs with the primary function of plant resource acquisition (Wardle et al. 2004; Huang et al. 2010; Mommer and Weemstra 2012; Osnas et al. 2013). The quantification of trait syndromes between the two organs is critical to understanding different strategies for plant resource acquisition (Withington et al. 2006; Fortunel et al. 2012) and could greatly increase our ability to recognize linkages between above‐ and belowground subsystems (Wardle et al. 2004; Sloan et al. 2013; Poorter et al. 2014).

Both specific leaf area (SLA) and specific root length (SRL) reflect organ potential growth rates associated with the resource capture of leaves and roots, respectively (Craine et al. 2001; Withington et al. 2006). The mass‐based N (nitrogen) concentration reflects the ability of plants to capture resources through enzymatic processes, and the mass‐based P (phosphorus) concentration determines the energy available for plant metabolisms (Güsewell 2004; Ågren 2008). The whole‐plant economic spectra clearly indicated that fast‐growing species tend to have high SLA, and SRL, as well as high N and P concentrations in both leaves and fine roots (Reich 2014). The accumulated evidence indicates that leaves and roots may express common strategies for resource acquisition (Díaz et al. 2004; Freschet et al. 2010; Liu et al. 2010) and that functional traits observed on leaves may, in part, be extrapolated to corresponding root traits (Tjoelker et al. 2005; Fort et al. 2012). Previous experiments shown the intra‐ and interspecific relationships of corresponding leaf and root traits, yet their results were frequently contradictory. For example, consistent patterns of thickness, tissue density (Craine et al. 2001; Westoby et al. 2013), N concentration (Craine and Lee 2003; Hajek et al. 2012; Chen et al. 2013), and resource acquisition trait, that is, SLA vs SRL (Withington et al. 2006; Hajek et al. 2012) have been found between leaves and fine roots, but many inconsistencies in these and other relationships, such as longevity, have also been found within an ecosystem and among different ecosystem types (Ruess et al. 1998; Tjoelker et al. 2005; Kembel and Cahill 2011; Fortunel et al. 2012; Freschet et al. 2013).

Plant height and root system size are thought to reflect the plant's ability to compete and survive. Large heights indicate a plant's capacity to successfully obtain aboveground resources (Price et al. 2014), and deep root systems are associated with the plant's capacity to successfully acquire water and nutrients from deeper soil layers (Fort et al. 2012). Therefore, plant height growth and root system size may be coupled, reflecting syndromes of above‐ and belowground morphology or size. Some evidence indicates that root and leaf traits are linked to the plant's potential growth rate (Comas et al. 2002). Maximum plant height and leaf functional traits represent independent axes upon which natural selection acts within some communities (Ackerly 2004; Price et al. 2014). On a global scale, however, individual leaf area shows a modest increase with plant height (Price et al. 2014). Compared with leaf traits, far less is known about root system traits (Mommer and Weemstra 2012), and it is as yet unclear whether and how plants of different heights would vary in root system size, especially in woody species.

The relationships between leaf and fine‐root traits are often related to broad selective pressures at different scales, giving rise to selective gradients for whole‐plant strategies (Liu et al. 2010; Kembel and Cahill 2011; Price et al. 2014). Plants growing within a single habitat are subjected to a greater number of similar selective pressures than plants across habitats (Price et al. 2014). Therefore, within a habitat, there can be strong relationships among leaf and root traits (Craine and Lee 2003). For instance, a significant positive SLA–SRL relationship has been found among temperate trees in central Poland (Withington et al. 2006). Liu et al. (2010) also showed coordinated variations in corresponding leaf and root traits (SLA vs. SRL, leaf versus root N and P concentrations) within a biome. Yet, the geographic area and associated differences in resource availability can influence the leaf–root trait relationships. Previous studies observed coordinated variations in leaf and root traits along environmental gradients, but the specific patterns of these relationships differed depending on the particular environmental gradient studied, such as the moisture vs. nutrient gradients of the arid ecosystem in northern China (Liu et al. 2010) or in the grasslands of New Zealand (Craine and Lee 2003). These studies on leaf–root trait relationships have often used measurements from across biomes (Craine and Lee 2003; Craine et al. 2005; Liu et al. 2010; Fort et al. 2012; Freschet et al. 2013), leading to confounding of the environmental effects driving the observed relationships. To better address these questions, a common garden experiment was used to study relationships intrinsic to the plants growing under similar environment selects (Wardle et al. 2004; Kembel and Cahill 2011; Poorter et al. 2014).

In this study, 23 deciduous and xerophytic woody species, including 3 tree species, 18 perennial shrubs, and two perennial subshrubs, were monitored in replicated, monoculture plots in a common garden experiment. All species come from the same ecosystem in the arid valley of the eastern Tibetan Plateau and undergone similar environmental selective pressures. Our main goals were to quantify trait relationships between leaves and fine roots relevant to their resource acquisition and to address the relationship between plant height growth rate and root system size. For the whole‐plant economics spectrum to hold true, we tested the two following predictions: (1) within a common condition, having nutrient‐rich, noncompletive and r‐environment selects, leaf, and fine‐root traits relevant to resource acquisition would be strongly coordinated across species. Species with leaves exhibiting high SLA, and leaf N and P concentrations would also exhibit parallel trait syndromes in fine roots. (2) Interspecific variations in the plant height growth should be tightly related to their root system size. Species with deep root systems should have improved plant growth and competitive capacities for aboveground resources, owing to their ability to obtain resources and better support aboveground organ survival (Fort et al. 2012).

## Materials and Methods

### Common garden experiment and species studied

Our field site was a common garden planting at the MMERS (Maoxian Mountain Ecosystem Research Station), Chinese Academy of Sciences, which is located in Maoxian County, Sichuan, China (31°41′ N, 103°53′ E; Alt. 1830 m). The climate of the site is temperate semiarid. Monthly climatic data, including mean rainfall, air temperature, evapo‐transpiration, air humidity, and hours of sunshine during this study period, were provided by the MMERS Meteorological Observatory. This site experiences an annual precipitation 738.8 mm and has a raining season running from May through October. The annual mean temperature is 10.4°C, with maximum temperatures of 18.7°C in July and minimum temperatures of –1.4°C in January. Annual sunshine time, mean air humidity, and evaporation were 1317.8 h, 75%, and 1018.2 mm, respectively. The ground slope across the common garden is <10°. The garden soil is characterized as Udic luvisols with uniform physical and chemical properties over the entire field. The 0–20 cm soil layer was characterized by 17.22 ± 2.02 g kg^−1^ organic matter, 1.64 ± 0.12 g kg^−1^ total N, 9.19 ± 1.45 mg kg^−1^ available P, and 8.25 ± 9.01 mg kg^−1^ available K, at pH 5.45 ± 0.11, with a 0.97 ± 0.10 g cm^−3^ bulk density. The soil water content was ~18% during the raining reason and ~7% during the dry reason.

This study used 1‐year‐old seedlings of 23 deciduous and xerophytic woody species. Fourteen families were included in this study. A full list of the observed species is shown in Appendix S1. The three tree species, *Ailanthus altissima* (Mill.) Swingle, *Koelreuteria paniculata* Laxm., and *Robinia pseudoacacia* Linn., are commonly used in the ecological restoration of dryland areas in China, and the 18 shrub and 2 subshrub species are native to the arid valley of the eastern Tibetan Plateau. Seeds of each species were collected from their natural habitats in the arid valley of the eastern Tibetan Plateau (31°42′N, 103°53′E, and an altitude range of 1600–1920 m) in autumn 2008. After natural air drying for 4–8 days, seeds of the *Rosaceae* species were scarified with 98% H_2_SO_4_ for 4 h and then were stratified at 5°C for 8 weeks to break seed dormancy. Other seeds were stored at room temperature (10–25°C) until sowing. Before sowing, all the seeds were disinfected by immersion in 2.5% NaClO for 1 h. All species were grown in the garden beginning in the spring of 2009.

The experimental design was a randomized block design with each of the 23 species replicated in five blocks per species. We established 15 plots (4 m × 4 m) for the tree species and 100 plots (2 m × 2 m) for the shrub and subshrub species. For each species, 20 seeds of a similar size were sown in each of the five plots on 28 March 2009. Shortly after emergence, seedlings of all the species were thinned and 10 plants per plot were retained on May 20. The distance between the plants was ~60–80 cm in each plot. The plots were hand‐watered and shaded uniformly until seedlings were established (about 1.5 month after sowing). In this way, we ensured that seedlings were not affected by drought and radiation stress. The garden was not fertilized during the experiment, but was manually weeded twice each year to reduce competition from other plants.

### Measurement of leaf and fine‐root traits

The leaves and roots of 115 plants of 23 woody species were collected at the end of the growing season (on 13 October 2009). We selected one plant with medium plant height and branch number in each of the five plots. To calculate the SLA, leaf area/leaf dry mass, 10–30 mature leaves of each plant were collected. Images of the leaves were recorded using a scanner (Model F6580, Founder Electronics Co Ltd, Beijing), and leaf areas were calculated with Image J 1.45 days (National Institute of Health, Washington DC). Leaves were dried in an oven for 48 h at 70°C for dry mass measurements.

Because the seedlings have small root systems and individual plants were separated from each other in each plot, an excavating method was used in this study. To measure the intact root systems of whole individuals, we unearthed one plant with medium plant height and branch number in each species and estimated their maximum rooting depths and widths prior to harvest. For each individual examined, an intact root system with soil was excavated and 95% RD (root depth) was estimated following the protocol of Cornelissen et al. (2003). All root samples with soil were detached from shoots, and kept cool and moist in iceboxes until cleaning and further processing in the laboratory. These root systems were carefully removed from the soil with forceps and gentle washing. All root branches of diameters <1 mm were defined as “fine roots”, and these were clipped from larger root branches and thoroughly cleaned of soil using a 0.5 mm (diameter) mesh sieve. Each fine‐root system was suspended in 1 cm (depth) of water and scanned (Epson EU‐88, Seiko Epson Corp. Japan). The TRL (total root length) of each subsample was measured using the WinRHIZO Pro 2007 software (Régent Instruments, Quebec, Canada). Fine roots were dried in an oven for 48 h at 70°C for dry mass measurements. The SRL was calculated as fine‐root length/fine‐root dry mass.

### Measurements of plant height growth, biomass, and root sizes

The plant height growth of each selected plant was measured using a linear scale from the stem base to the top of the branches before harvesting on 13 October 2009. Once plants were harvested, shoots of each plant were divided into stems and leaves. The dry mass was determined for the same segment. During the experimental period, senesced and dropped leaves from all individuals were collected for total leaf dry mass determinations. The total plant BM (biomass) was calculated as the sum of root, stem, and leaf masses.

### Element concentration analysis

All dried fine roots and leaves were ground and homogenized manually in a mixer mill (SPEX 8000‐D, Edison, NJ). The total N concentrations were determined using high temperature combustion in a Vario Macro Cube Elemental Analyser (Elementar Analysensysteme GmbH, Germany). The total phosphorus concentrations were analyzed using colorimetry after digestion with H_2_O_2_–H_2_SO_4_.

### Statistical analyses

We calculated the means and standard errors of all of the traits for each species (*n* = 5) and tested relationships between traits using species traits means. Appendix S1 shows the means and standard errors of all the variables in this study. A PCA (principal component analysis) was performed to determine the relationships both within and among the corresponding traits of leaves and fine roots for the 23 species and to determine plant trait syndromes using R software (Version 3.13.0, R Development Core Team, 2014). The trait‐by‐trait interrelationships were then tested using a Pairwise Pearson Correlation. Finally, significant relationships between corresponding leaf and root traits, such as SLA vs. SRL, and leaf versus root N and P concentrations and between root traits and plant relative growth rates among species were analyzed using linear regressions. Significance tests were conducted using SPSS (Version 16.0, SPSS Inc., Chicago, IL). The plant functional types were tree, shrub, and subshrub. As the tree and subshrub groups were comprised of only three and two species, respectively, the statistical analyses were made across all 23 species without accounting for plant functional type.

PICs (Phylogenetic independent contrasts) were performed in the R software (using the picante package). The phylogenetic tree for 23 species was constructed using the Phylomatic utility, based on APG III and Flora of China. PICs were also performed to analyze the evolutionary relationships between a set of traits.

## Results

### Plant trait syndromes

**Figure 1:**
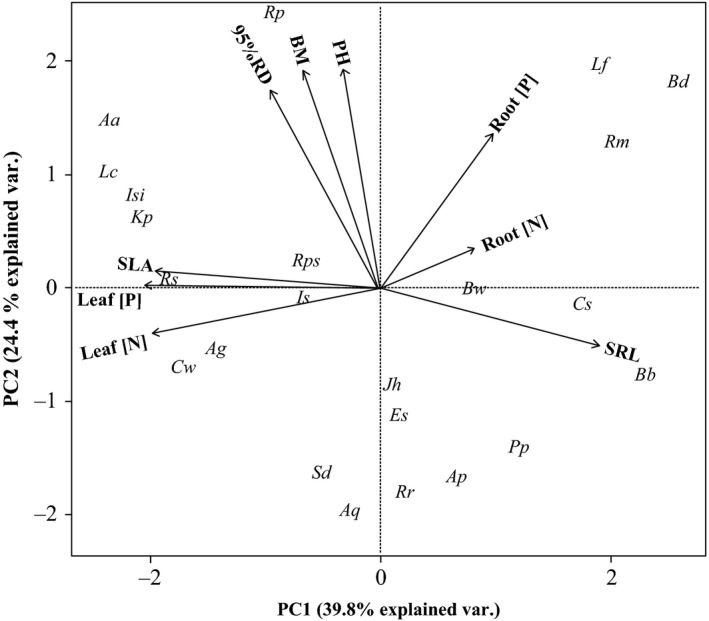
Display of species traits along the first two principal component analysis axes. Species abbreviation (italics) is given in Appendix S1. BM, biomass; PH, plant height, 95%RD, 95% of root depth; TRL, total fine‐root length; SRL, specific fine‐root length; Root [N], fine‐root nitrogen concentration; Root [P], fine‐root phosphorus concentration; SLA, specific leaf area; Leaf [N], leaf nitrogen concentration; and Leaf [P], leaf phosphorus concentration.

The first two axes of the PCA explained ~63% (Axis 1: 40.9%; Axis 2: 21.8%) of the variability (Fig. 1). The first axis was defined by the coordinated physiological and chemical traits of both fine roots and leaves. These traits were divided into two significantly but negatively correlated groups. One leaf‐related group, including SLA, and leaf–root N and P concentrations, negatively correlated with Axis 1. Another root‐related group, including SRL, and fine N and P concentrations, positively correlated with Axis 1. Therefore, the main trends of fine root and leaf interspecific variation separated species with small SLA and low element concentrations in leaves, and great SRL and high element concentrations in fine roots, from those with the opposite sets of traits. The second PCA axis was driven by interspecific variations in plant size (Fig. 1), and thus, represents a trend from dwarf species with shallow roots to tall species with deep roots.

### Leaf and fine‐root trait relationships

**Figure 2:**
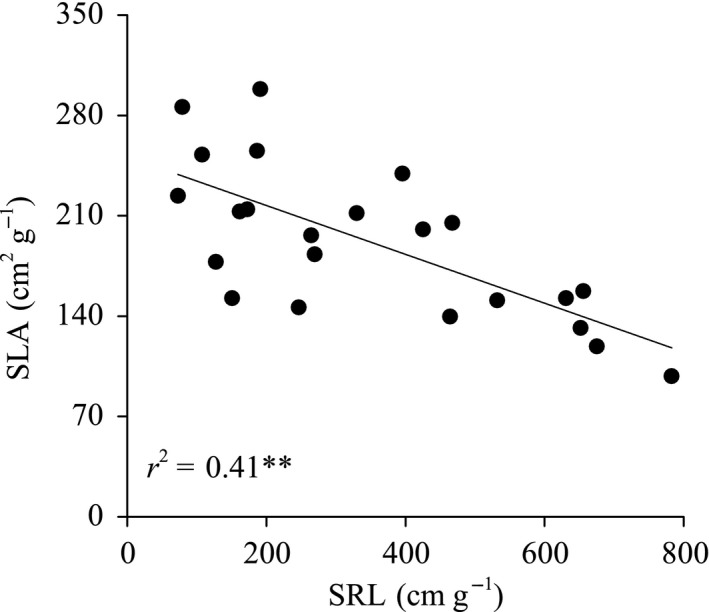
Relationship between specific leaf area and specific root length for 23 woody species from the arid valley of the eastern Tibetan Plateau. **P <* *0.01.

SLA was negatively correlated with SRL (*n* = 23, *r* ^2^ = 0.41; *P *<* *0.01; linear regression; Fig. 2), indicating that species with smaller SLA have greater SRL than species with greater SLA. At the same time, leaf N and P concentrations were not related to corresponding root nutrient concentrations (Table 1).

**Table 1:** Pearson correlation coefficients and significances among plant BM (biomass), plant height (PH), and the root and leaf traits for all 23 species from the arid valley of the eastern Tibetan Plateau. Below diagonal is Pearson correlation analyses, and above diagonal is based on phylogenetically independent contrasts

	BM	PH	95%RD	TRL	SRL	Root[N]	Root[P]	SLA	Leaf[N]	Leaf[P]
BM		0.736**	0.454*	0.429*	0.044ns	−0.195ns	0.355ns	0.062ns	−0.071ns	0.134ns
PH	0.724**		0.810**	0.484*	−0.212ns	−0.282ns	0.278ns	0.322ns	0.290ns	0.426*
95%RD	0.679*	0.590**		0.393ns	−0.554**	−0.357ns	0.038ns	0.685**	0.640**	0.692**
TRL	0.554**	0.637**	0.292		−0.006ns	−0.329ns	−0.164ns	0.102ns	0.007ns	0.160ns
SRL	−0.109ns	0.132ns	−0.541*	−0.036ns		0.301ns	0.473*	−0.849**	−0.707**	−0.783**
Root[N]	−0.425*	−0.494*	−0.174ns	−0.324ns	0.076ns		0.615**	−0.270ns	−0.153ns	−0.378ns
Root[P]	0.038ns	−0.035ns	0.153ns	−0.123ns	0.252ns	0.660**		−0.334ns	−0.376ns	−0.378ns
SLA	0.436*	0.080ns	0.646**	0.115ns	−0.709**	−0.161ns	−0.159ns		0.622**	0.671**
Leaf[N]	0.195ns	0.036ns	0.35ns	−0.227ns	−0.580**	0.099ns	−0.244ns	0.433*		0.797**
Leaf[P]	0.292ns	0.028ns	0.513**	0.141ns	−0.757**	−0.181ns	−0.352ns	0.633**	0.508**	

ns, nonsignificant.

**P <* *0.01, *P <* *0.05.

### Relationships between above‐ and belowground plant sizes

**Figure 3:**
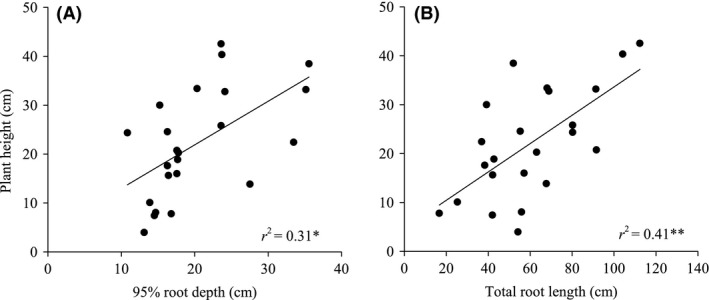
Relationships of the plant height to 95% root depth and to total root length for 23 woody species collected from the arid valley of the eastern Tibetan Plateau and grown in a common garden in Maoxian, Sichuan, China. *P <* *0.05; **P <* *0.01.

Species with a deep and thick root system (95% RD and TRL) generally had higher plant height growth values than species with shallow root systems (*n* = 23, *r* ^2^ = 0.63, *P *<* *0.01; linear regression, Fig. 3). However, there were no significant relationships between biomass and root system size (Table 1).

## Discussion

Our work tested a long‐held belief that leaf and root traits relevant to resource acquisition are coordinated across species (Grime 2001; Freschet et al. 2010; Fortunel et al. 2012; Fort et al. 2012; Reich 2014). SLA, SRL, and plant growth rates have been previously found to positively relate to each other in the global whole‐plant economic spectrum (Comas et al. 2002; Osnas et al. 2013; Westoby et al. 2013; Reich 2014) and in regional tropical and temperate forests (Withington et al. 2006; Li et al. 2010; Hajek et al. 2013; Fort et al. 2012). However, these general positive relationships between SLA and SRL are inconsistent in many grassland systems (Tjoelker et al. 2005; Kembel and Cahill 2011). In contrast to the results found in forests and grasslands, we found a negative relationship between leaf and root traits critical to resource acquisition for seedlings of xerophytic woody species observed in a common environment (Fig. 1). This result did not provide support for our first hypothesis of broadly coordinated variations across species for the aboveground and belowground resource acquisition traits.

Explanations of the leaf–root trait relationships have focused on the role of environmental factors in different habitats, giving rise to selection gradients for whole‐plant functional strategies. However, the selective pressure can influence above‐ and belowground traits differently (Craine and Lee 2003; Craine et al. 2005; Liu et al. 2010). For example, the leaf and root traits were positively correlated within biomes, but negatively correlated among the temperate grassland biomes of Alberta (Kembel and Cahill 2011). Our results demonstrated different resource acquisition traits between leaves and fine roots when plants face a drought environment select. Leaves are deactivated during drought to reduce the consumption of water and nutrient (Gargallo‐Garriga et al. 2014), reflecting by small and thick leaves with small SLA (Teklehaimanot et al. 1998; Wright et al. 2004; Reich 2014), whereas roots are activated to enhance the uptake of water and nutrients, reflecting by the thin roots with great SRL (Eissenstat 1991; Metcalfe et al. 2008; Leva et al. 2009; Chen et al. 2013). This led to a negative relationship between SLA and SRL across species, that is, species with small SLA matching with great SRL. The inverse pattern of leaf and root traits may be a whole‐plant strategy for favorable resource acquisition in these xerophytic species. This finding is consistent with the physiological properties of seminatural grasslands (Gargallo‐Garriga et al. 2014), which also suggest different metabolomes, and nutrient and elemental stoichiometries among shoots and roots, as well as the inverse physiological responses of shoots and roots to drought. Liu et al. (2010) suggested that the SLA to SRL ratio decreased as the aridity index increased in desert area of the northern China. More research is necessary to test this relationship in other arid regions and other woody species.

Consistent above‐ and belowground nutrient (N and P) concentration relationships were found by others in grassland and savannah ecosystems (Craine and Lee 2003; Craine et al. 2005; Tjoelker et al. 2005), arid areas in northern China (Liu et al. 2010), and temperate and tropical forests (Li et al. 2010). There is a general positive relationship between leaf and root N concentrations in these ecosystems. Unlike these results, we found an independent pattern of variations in leaf and root N and P concentrations among species when grown in a common garden set. The concentrations of both nutrients were unrelated between leaves and roots across species (Table 1).

This lack of chemical trait syndromes may be due to the different physiological processes in leaves and roots (Chen et al. 2013; Gargallo‐Garriga et al. 2014). The results from temperature forest had demonstrated that nutrient concentrations of leaves are related to the photosynthetic capacity influencing by Rubisco's concentration or activity, whereas nutrients of fine roots are absorbed by the root surface directly and by mycorrhizal fungi (Chen et al. 2013). Evidence for the independent patterns of nutrient concentrations among leaves and roots is lacking because there are few datasets available to test leaf–root nutrient relationships collected from the same sets of plants. Tjoelker et al. (2005) suggested that a strong positive relationship in N concentration between leaves and fine roots partly due to nutrient stress in grassland. Alternatively, the weak leaf and fine‐root traits relationship with N and P concentrations shown by seedlings in the present study perhaps reflects nutrient‐sufficient environments (1.64 ± 0.12 g kg^−1^ total N, 9.19 ± 1.45 mg kg^−1^ available P). The results of the present study are in agreement with earlier finding (Kembel and Cahill 2011) and suggest that the coordinated patterns of leaf and root traits, particularly in relation to nutrient economics, are not necessarily widespread. For example, variations in leaf and root N concentrations were positively related at inter‐ and intraspecific levels, while leaf and root P concentrations were not (Li et al. 2010; Freschet et al. 2013).

Our result opposed the generally accepted notion that a deep root system is a conservative trait associated with a slow total plant growth rate (Eissenstat et al. 2000; see Wright et al. 2004). There was a strong positive correlation between plant height growth and root system size in the present study (Fig. 3). This result provides support for our second hypothesis and reflects the fundamental trade‐offs in morphology and size between above‐ and belowground plant organs. During seedling establishment, the fast growth rate of aboveground organs and whole plants requires a rapid resource uptake by the belowground organs (Comas et al. 2002). As such, these deep root systems increase a plant's ability to uptake water from deep soil layers, obtaining enough resources for the aboveground organs survival. This allows the plant to continue plant height growth and improves the competitive capacity for aboveground resources (Fort et al. 2012).

Although species with deep root systems had smaller SRL and greater plant height, there was no relationship between plant height and SRL across species (Table 1). Similar to our study, Comas et al. (2002) found that slow‐growing species have smaller SRL in the seedlings of temperate tree species, but no relationship existed between shoot growth rate and root morphology (SRL and diameter). In contrast, comparisons between grass species indicated that slow‐growing species from stressful habitats have deep, yet coarser root system than fast‐growing species (Fort et al. 2012). Whether this is a general difference in plant strategy between woody plants and grasses is unclear. The ontogenetic variations of these relationships need to be examined.

## Conclusions

In this common garden experiment, we found that there were often negative or nonsignificant relationships between many corresponding leaf and root traits, which contrasted the prevailing expectation of consistent positive relationships among leaf and fine‐root traits. These results provide limited support for the hypothesis that resource acquisition is coordinated among above‐ and belowground plant organs as part of a whole‐plant resource acquisition strategy. Our results highlight that inverse or independent leaf–root relationships exist across species and reveal that there may be different resource acquisition strategies between above‐ and belowground plant organs, even when they are subjected to similar selective pressures. These findings provide a new understanding of above‐ and belowground subsystems in carbon and economy and their interactions. They also suggest that the resource acquisition observed in leaves cannot be extrapolated to predict root structures in many systems.

## Conflict of Interest

None declared.

## Supporting information


**Appendix S1.** The studied 23 plant species from the arid valley of the eastern Tibetan Plateau, their growth forms (following http://foc.eflora.cn/), plant total biomass (BM), Plant height (PH), 95% root depths (95% RD), total fine root lengths (TRL), specific root lengths (SRL),fine root nitrogen concentrations (Root [N]), fine root phosphorus concentrations (Root [P]), specific leaf area (SLA), leaf nitrogen concentration (Leaf [N]) and leaf phosphorus concentrations (Leaf [P]). Values are means of five individuals.
