# Supplementary material for: New insights into leaf and fine‐root trait relationships: implications of resource acquisition among 23 xerophytic woody species
Source: Ecol Evol. 2015 Oct 29;5(22):5344–51. doi: 10.1002/ece3.1794 (PMC6102526; doi:10.1002/ece3.1794)
Supplement: Supplementary file 1 — Appendix S1. The studied 23 plant species from the arid valley of the eastern Tibetan Plateau, their growth forms (following http://foc.eflora.cn/), plant total biomass (BM), Plant height (PH), 95% root depths (95% RD), total fine root lengths (TRL), specific root lengths (SRL),fine root nitrogen concentrations (Root [N]), fine root phosphorus concentrations (Root [P]), specific leaf area (SLA), leaf nitrogen concentration (Leaf [N]) and leaf phosphorus concentrations (Leaf [P]). Values are means of five individuals. [file ECE3-5-5344-s001.docx]

**Appendix 1** The studied 23 plant species from the arid valley of the eastern Tibetan Plateau, their growth forms (following http://foc.eflora.cn/), plant total biomass (BM), Plant height (PH), 95% root depths (95% RD), total fine root lengths (TRL), specific root lengths (SRL),fine root nitrogen concentrations (Root [N]) , fine root phosphorus concentrations (Root [P]), specific leaf area (SLA), leaf nitrogen concentration (Leaf [N]) and leaf phosphorus concentrations (Leaf [P]). Values are means of five individuals.

| Specie | Abbr. | Family | Life form | BM  (g) | PH  (cm) | 95%RD  (cm) | TRL  (cm) | SRL  (cm g^-1^) | Root[N]  (mg g^-1^) | Root[P]  (mg g^-1^) | SLA  (cm^2^ g^-1^) | Leaf N]  (mg g^-1^) | Leaf[P]  (mg g^-1^) |
| --- | --- | --- | --- | --- | --- | --- | --- | --- | --- | --- | --- | --- | --- |
| *Ailanthus altissima*(Mill.) Swingle | *Aa* | Simaroubaceae | Tree | 9.34±0.45 | 13.8±0.9 | 27.5±04 | 67.7±10.8 | 79.1±8.8 | 11.6±0.5 | 2.59±0.12 | 286.2±9.0 | 34.4±0.3 | 2.30±0.08 |
| *Ajaniapotaninii*(Krasch.) Poljak | *Ap* | Asteraceae | Subshrub | 1.24±0.07 | 24.4±0.3 | 10.8±0.2 | 80.2±5.1 | 655.7±74.8 | 10.3±0.6 | 1.51±0.04 | 157.7±3.7 | 27.6±0.1 | 2.42±0.04 |
| *Akebiaquinata* (Thunb.) Decne. | *Aq* | Lardizabalaceae | Shrub | 1.17±0.25 | 7.5±0.2 | 14.5±0.8 | 42.0±7.7 | 395.8±20.6 | 12.2±0.6 | 1.87±0.01 | 239.5±3.2 | 28.3±1.4 | 2.65±0.02 |
| *Artemisia gmelinii* Web.et Stechm | *Ag* | Asteraceae | Subshrub | 2.91±0.15 | 24.6±1.5 | 16.3±1.1 | 55.3±8.3 | 329.6±22.4 | 9.5±0.3 | 1.84±0.05 | 211.9±3.7 | 30.8±0.6 | 3.34±0.10 |
| *Bauhinia brachycarpa* var. *microphylla* | *Bb* | Fabaceae | Shrub | 4.56±0.63 | 30.0±1.9 | 15.2±0.9 | 39.2±6.0 | 783.0±69.6 | 14.0±0.5 | 3.03±0.03 | 98.20±2.8 | 28.5±0.5 | 2.17±0.02 |
| *Berberiswilsonae* | *Bw* | Berberidaceae | Shrub | 3.29±0.22 | 20.8±0.7 | 17.6±0.4 | 91.6±10.8 | 464.3±36.4 | 16.4±0.8 | 2.59±0.06 | 140.0±7.0 | 23.1±0.4 | 2.93±0.05 |
| *Buddlejadavidii* Fr. | *Bd* | Loganiaceae | Shrub | 3.91±0.29 | 33.4±2.2 | 20.3±0.5 | 68.1±9.9 | 675.3±53.4 | 20.6±0.4 | 3.99±0.05 | 118.7±1.1 | 25.7±0.1 | 2.21±0.10 |
| *Ceratostigmawillmottianum*Stapf ex Prain | *Cw* | Plumbaginaceae | Shrub | 8.91±0.98 | 40.4±0.3 | 23.7±0.3 | 104.2±8.9 | 127.3±13.9 | 7.4±0.4 | 1.81±0.11 | 178.0±3.3 | 29.7±1.8 | 3.06±0.32 |
| *Cotinusszechuanensis* A. Penze | *Cs* | Ranunculaceae | Shrub | 2.65±0.13 | 17.6±0.9 | 16.3±0.6 | 38.3±5.5 | 631.0±37.2 | 12.3±0.2 | 3.23±0.10 | 152.9±5.1 | 21.5±0.6 | 2.29±0.09 |
| *Elaeagnusstellipila*Rehd | *Es* | Elaeagnaceae | Shrub | 1.44±0.14 | 8.0±0.2 | 14.7±0.3 | 55.9±1.8 | 467.5±33.6 | 28.1±0.5 | 3.19±0.05 | 205.4±7.4 | 37.2±0.5 | 2.63±0.18 |
| *Indigoferasilvestrii*Pamp. | *Isi* | Fabaceae | Shrub | 5.91±0.45 | 25.8±1.9 | 23.6±0.3 | 80.3±7.4 | 161.5±33. 3 | 19.3±0.9 | 2.70±0.12 | 213.0±5.4 | 37.5±0.7 | 3.49±0.15 |
| *Indigoferaszechuensis*Craib | *Is* | Fabaceae | Shrub | 2.20±0.13 | 16.0±1.2 | 17.6±0.6 | 57.2±3.3 | 172.5±6.9 | 22.0±1.2 | 2.89±0.11 | 214.5±1.1 | 31.8±0.4 | 2.81±0.03 |
| *Jasminumhumile* Linn. | *Jh* | Oleaceae | Shrub | 2.16±0.13 | 18.9±0.9 | 17.7±0.1 | 42.7±7.1 | 246.5±41.5 | 13.9±0.5 | 1.59±0.01 | 146.3±5.0 | 28.6±1.0 | 2.51±0.09 |
| *Koelreuteriapaniculata*Laxm. | *Kp* | Sapindaceae | Tree | 11.40±0.01 | 38.5±0.8 | 35.6±0.2 | 52.0±6.3 | 191.0±12.4 | 13.6±0.2 | 2.83±0.09 | 298.5±14.9 | 39.7±1.4 | 3.01±0.20 |
| *Lespedeza floribunda* Bunge | *Lf* | Fabaceae | Shrub | 5.22±0.25 | 15.7±1.7 | 16.4±0.3 | 42.1±5.3 | 532.2±21.0 | 26.2±1.0 | 4.71±0.11 | 151.0±4.3 | 20.6±0.6 | 2.10±0.03 |
| *Lyciumchinense* Mill. | *Lc* | Solanaceae | Shrub | 3.17±0.34 | 22.4±0.2 | 33.5±0.2 | 36.9±5.7 | 72.6±13.3 | 20.9±0.3 | 2.91±0.04 | 223.9±7.7 | 38.3±2.1 | 3.35±0.34 |
| *Podocarpium podocarpum* (DC.) Yang et Huang | *Pp* | Fabaceae | Shrub | 0.94±0.05 | 4.0±0.1 | 13.1±0.2 | 54.2±8.5 | 269.4±38.8 | 26.0±0.5 | 3.47±0.06 | 183.2±4.2 | 26.2±0.2 | 2.34±0.13 |
| *Rhamnusrosthornii*Pritz. | *Rr* | Rhamnaceae | Shrub | 1.16±0.86 | 7.8±0.7 | 16.8±0.1 | 16.7±3.7 | 264.4±33.3 | 16.7±0.4 | 1.79±0.01 | 196.4±2.0 | 39.4±0.3 | 3.11±0.05 |
| *Robiniapseudoacacia* Linn. | *Rp* | Fabaceae | Tree | 5.70±0.37 | 33.2±0.3 | 35.2±0.3 | 91.3±7.7 | 186.3±16.9 | 14.3±0.2 | 4.31±0.19 | 255.5±3.6 | 28.4±0.1 | 2.97±0.08 |
| *Rosamultibracteata*Hemsl. et Wils. | *Rm* | Rosaceae | Shrub | 1.16±0.68 | 20.3±7.1 | 17.8±0.2 | 63.1±12.4 | 652.1±26.8 | 13.7±0.2 | 3.16±0.09 | 132.0±1.7 | 25.5±0.7 | 2.01±0.04 |
| *Rosa soulieeana* Crép. | *Rs* | Rosaceae | Shrub | 5.33±0.34 | 32.8±0.4 | 24.1±0.7 | 69.0±10.9 | 107.3±2.5 | 7.5±0.5 | 1.42±0.04 | 252.7±15 | 27.5±0.0 | 2.85±0.11 |
| *Rubuspungens*Camb. var. *ternatus* Card. | *Rps* | Rosaceae | Shrub | 12.29±0.29 | 42.6±0.4 | 23.6±0.7 | 112.4±12.4 | 425.5±89.1 | 9.7±1.3 | 1.51±0.13 | 200.8±3.1 | 27.5±0.2 | 2.59±0.04 |
| *Sophoradavidii* (Franch.) Skeels | *Sd* | Fabaceae | Shrub | 1.40±0.03 | 10.1±0.5 | 13.9±0.3 | 25.4±1.9 | 150.6±5.6 | 15.6±0.3 | 2.49±0.08 | 152.6±7.0 | 37.5±0.5 | 2.71±0.19 |
